# The Impact of Sample Size and Population History on Observed Mutational Spectra: A Case Study in Human and Chimpanzee Populations

**DOI:** 10.1093/gbe/evad019

**Published:** 2023-02-15

**Authors:** Suhail Ghafoor, João Santos, Cyril J Versoza, Jeffrey D Jensen, Susanne P Pfeifer

**Affiliations:** Center for Evolution and Medicine, Arizona State University, Tempe, AZ, USA; School of Life Sciences, Center for Evolution and Medicine, Arizona State University, Tempe, AZ, USA; School of Life Sciences, Center for Evolution and Medicine, Arizona State University, Tempe, AZ, USA; School of Life Sciences, Center for Evolution and Medicine, Arizona State University, Tempe, AZ, USA; School of Life Sciences, Center for Evolution and Medicine, Arizona State University, Tempe, AZ, USA

**Keywords:** mutation, mutation spectrum, demographic history, sampling effect

## Abstract

Recent studies have highlighted variation in the mutational spectra among human populations as well as closely related hominoids—yet little remains known about the genetic and nongenetic factors driving these rate changes across the genome. Pinpointing the root causes of these differences is an important endeavor that requires careful comparative analyses of population-specific mutational landscapes at both broad and fine genomic scales. However, several factors can confound such analyses. Although previous studies have shown that technical artifacts, such as sequencing errors and batch effects, can contribute to observed mutational shifts, other potentially confounding parameters have received less attention thus far. Using population genetic simulations of human and chimpanzee populations as an illustrative example, we here show that the sample size required for robust inference of mutational spectra depends on the population-specific demographic history. As a consequence, the power to detect rate changes is high in certain hominoid populations while, for others, currently available sample sizes preclude analyses at fine genomic scales.

**Significance**

Gaining a better understanding of rates and patterns of mutation is central to evolutionary biology. Comparative genomic analyses can help researchers to elucidate the causes of the variation observed among taxa; however, limited sample sizes pose several challenges for many species, including nonhuman primates. Here, we show that the number of samples required for robust inference of population-specific mutational spectra depends greatly on the organism's underlying demographic history, and importantly may thus be quantified prior to data collection and analysis.

## Introduction

As has long been appreciated, mutation is a fundamental force of molecular evolution. Importantly though, rates and patterns of mutation have themselves evolved across the tree of life as a by-product of, among other factors, variations in species-specific cellular and developmental processes, as well as differential environmental exposures to mutagens (see reviews by Baer et al. 2007; Pfeifer 2020). Although changes in the germline mutation spectra (i.e., the relative rate of point mutations accumulating in different local sequence contexts) across mammalian species have been well-documented for decades (e.g., Hwang and Green 2004), recent studies have, perhaps surprisingly, observed shifts in the frequency of particular mutation types over much shorter evolutionary time scales. For example, such a shift has been documented among human populations (Harris 2015; Harris and Pritchard 2017; Mathieson and Reich 2017; Narasimhan et al. 2017; Aikens et al. 2019). In particular, using the spectrum of population-specific segregating variants, Harris (2015) identified a substantial rate increase of a single mutation type, 5′ TCC 3′ → 5′ TTC 3′ (hereafter denoted as TCC > T) mutations, in European compared with African and, to a lesser extent, East Asian populations. Although this enrichment appears to be the most prominent signature in recent human history, subsequent studies, either focusing on different populations (Harris and Pritchard 2017; Mathieson and Reich 2017; Narasimhan et al. 2017) or wider genomic contexts (Aikens et al. 2019), identified several additional, albeit more subtle, changes in population-specific mutational spectra.

Differences in mutational spectra have also been observed among closely related hominoids (Harris and Pritchard 2017), likely caused by a combination of local compartment-specific (i.e., within specific genomic regions) and global species-specific changes (Goldberg and Harris 2022). Taken together, these results suggest that mutational signatures might, at least to some extent, be driven by fine-scale population-specific changes in the underlying cellular mutational processes, potentially due to the (temporary) presence of natural mutators (Seoighe and Scally 2017). In turn, the accurate characterization of these mutational inputs is vital for questions ranging from the timing of evolutionary events in these species to characterizing the relative contributions of adaptive versus nonadaptive processes in shaping levels and patterns of genomic variation (Pfeifer and Jensen 2016).

Yet, concerns have recently been raised that significant batch effects and/or sequencing errors (especially for polymorphisms segregating at low frequencies) might have led, or at least contributed, to some of the mutational shifts that have been observed (Anderson-Trocmé et al. 2020). However, other potentially confounding factors, such as the impact of sample size, have received less attention thus far. Although analyses pertaining to human populations benefit from the immense public resources provided by scientific consortia—such as the 1000 Genomes Project (including >2,500 individuals from 26 populations; 1000 Genomes Project Consortium 2015) and the Simons Genome Diversity Project (including ∼300 individuals from 142 populations; Mallick et al. 2016)—sample sizes in many nonhuman primates remain much more limited, particularly for those species not utilized in biomedical research. Specifically, the Great Ape Diversity Panel (Prado-Martinez et al. 2013) which is utilized by Harris and Pritchard (2017) to infer the great ape mutational spectra (and re-analyzed by Goldberg and Harris 2022) consists of 83 great ape genomes: 9 humans, 24 common chimpanzees (10 Nigerian-Cameroon, 6 Eastern, 4 Central, and 4 Western chimpanzees), 13 bonobos, 27 gorillas (3 Eastern lowland, 1 Cross river, and 23 Western lowland gorillas), and 10 orangutans (5 Sumatran and 5 Bornean orangutans). Moreover, although extensive population-specific differences have been observed in human mutational spectra, due to the limited sample sizes, several populations of nonhuman great apes were previously jointly analyzed (e.g., chimpanzees; see fig. 5 and supplementary fig. 1 in Harris and Pritchard 2017), despite known strong population structure (Fischer et al. 2011).

Exacerbating these issues is the observation that certain types of mutations can be sensitive to recent demography (see discussion in Mathieson and Reich 2017), which remains unaccounted for in many studies published to date. In fact, population history, which exerts a direct influence on genetic diversity, is known to vary profoundly among populations and species. Among humans, for example, many African populations are thought to have retained relatively stable effective population sizes (*N_e_*) throughout their history, whereas European and Asian populations have experienced rapid population growth after an initial out-of-Africa bottleneck, leading to an excess of population-specific rare variants (Gravel et al. 2011). In contrast, chimpanzee populations exhibit vastly different effective population sizes—ranging from ∼5,700 in Western chimpanzees to ∼72,000 in Central chimpanzees (Prado-Martinez et al. 2013), potentially leading to more (high *N_e_*) or less (low *N_e_*) efficient selection in removing mutator alleles from a population.

Revisiting previous analyses focusing on humans and chimpanzees as a case study (and as the best characterized representatives of the great apes), we hence here investigate the following questions: First, differences in study design and inevitable sequencing errors aside, how much variance in the mutational spectra can we expect from the limited sample sizes currently available for hominoids and, relatedly, how large of a sample size would be required to accurately reflect the mutation spectrum of the entire population? Second, taking into account the population-specific demographic histories, what is the magnitude of rate change that can reliably be inferred given a particular sample size and scale? Using population genetic simulations of previously inferred demographic models (Gutenkunst et al. 2009; Gravel et al. 2011; Prado-Martinez et al. 2013), we show that the number of samples required for robust inference of population-specific mutational spectra depends greatly on the underlying demographic history. Moreover, although the power to detect rate changes is high in certain populations; for others, the currently available sample sizes preclude analyses at fine genomic scales. Notably, although human and chimpanzee populations are here utilized as illustrative examples, this work speaks broadly to the inference of mutation rate information from population genomic data. We further highlight mutational spectra as an important (and potentially confounding) factor in evolutionary genomic analyses, and present a guide for how to assess the impacts of sample size and population history on such inference.

## Materials and Methods

### Simulations: Human and Chimpanzee Demographic Models

**Fig. 1.**
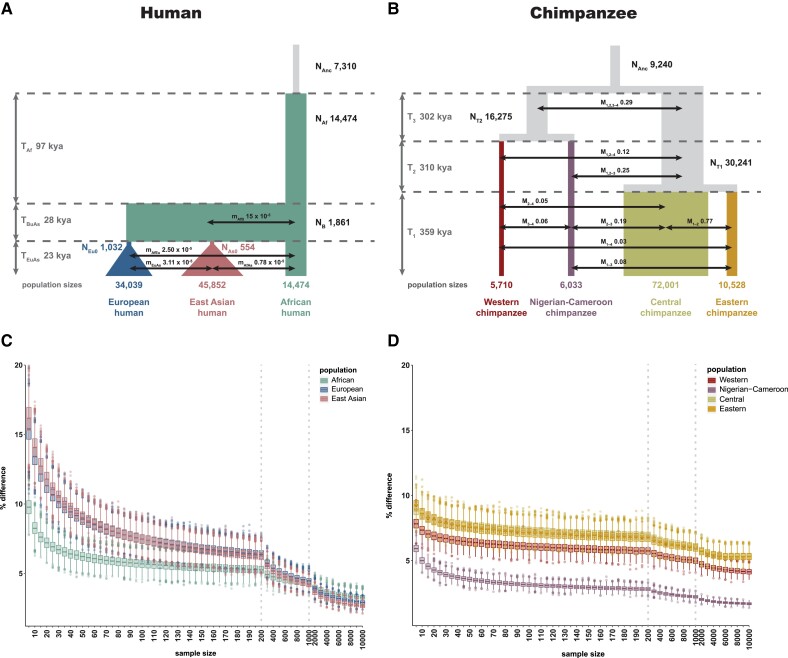
Effect of sample size and population demography on broad-scale mutational spectra. *Top panels:* (*A*) Human demographic history for European (blue), East Asian (pink), and African (turquoise) populations inferred by Gravel et al. (2011). (*B*) Chimpanzee demographic history for Western (red), Nigerian-Cameroon (purple), Central (green), and Eastern (yellow) populations inferred by Prado-Martinez et al. (2013). *N* = population size; *T* = split time; *M* = number of migrants; *m* = migration rate; kya = thousand years ago. *Bottom panels:* Comparison of the mutational spectra between whole populations of (*C*) human and (*D*) chimpanzee populations subsampled with replacement to (*i*) 5–200 individuals (in increments of 5), (ii) 300–1,000 individuals (in increments of 100), and (iii) 2,000–10,000 individuals (in increments of 1,000) at the broad (chromosomal) scale. Comparisons were performed by calculating the sum of the differences in the distributions of each mutation type between the subsamples and the whole population. Dotted lines indicate changes in subsampling scheme.

Data sets were simulated for three human (African, East Asian, and European) and four chimpanzee (Nigerian-Cameroon [*Pan troglodytes ellioti*], Eastern [*Pan troglodytes schweinfurthii*], Central [*Pan troglodytes troglodytes*], and Western [*Pan troglodytes verus*]) populations using SLiM v.3 (Haller and Messer 2019). Specifically, human data were simulated according to the demographic model initially introduced by Gutenkunst et al. (2009) and utilized by Gravel et al. (2011) (fig. 1*A*; for additional information, refer to the “Low-coverage + exons” model in their table 2 and fig. 4). Chimpanzee data were simulated according to the demographic model inferred by Prado-Martinez et al. (2013) (fig. 1*B*; and see model 4A in their supplementary fig. 12.3.4 and supplementary table 12.3.5). All simulations were based on previously inferred maximum likelihood parameter estimates (human) or maximum *a posteriori* probability parameter estimates (chimpanzee). Following previous work, generation times were converted to years assuming generation times of 25 years for human (Gravel et al. 2011) and 20 years for chimpanzee (Prado-Martinez et al. 2013).

### Simulations: Basic Demographic Models

To aid the interpretation of the results, three additional basic demographic models were simulated using SLiM v.3 (Haller and Messer 2019). The basic models start with an ancestral population size of 10,000 individuals. After a burn-in period of 10*N_e_* (100,000) generations, a population splits from the ancestral population to a size of 10,000 individuals. This subpopulation either stays at a constant size (model 1—“constant”) or experiences an instantaneous size change of one order of magnitude, either decreasing to 1,000 individuals (model 2—“decline”) or increasing to 100,000 individuals (model 3—“growth”). For each of the three basic demographic models, broad-scale mutational landscapes were assessed by simulating 1,000 replicates of a full-length chromosome (chromosome 21) under a custom mutational matrix model (supplementary fig. S1, Supplementary Material online, and see the next section for additional details).

### Simulations: Broad-scale (Global) Mutational Landscapes

For each species, 1,000 replicates of a full-length chromosome (chromosome 21) were simulated based on the nucleotide sequence obtained from the species-specific reference assembly available in NCBI GenBank (T2T-CHM12 v.2.0 for humans [accession number: CP068257; Nurk et al. 2022] and panTro6 for chimpanzees [accession number: CM009259; Kronenberg et al. 2018]). Missing sites present in the chimpanzee reference assembly were replaced by nucleotides (i.e., A, C, G, and T), taking into account their relative frequencies on the chromosome. Chromosomes were simulated using a mutational model based on previously inferred context-specific mutation rates for humans and chimpanzees (Harris and Pritchard 2017; Aikens et al. 2019), with an overall mutation rate of 10^−8^ per base pair per generation. Recombination was considered constant at a rate of 10^−8^ per base pair per generation, in accordance with previous estimates for the species (1000 Genomes Project Consortium 2010; Auton et al. 2012). Each of these 1,000 independent replicates represents one potential realization of the population-specific mutational spectra of the whole populations (see below). To assess the effect of sample size, each full population was subsampled with replacement to (1) 5–200 individuals (in increments of 5), (2) 300–1,000 individuals (in increments of 100), and (3) 2,000–10,000 individuals (in increments of 1,000), and five independent replicates were drawn for each subsample.

### Population-Specific Mutational Spectra

To mimic the analysis of empirical data, mutational spectra were calculated based on population-specific biallelic single-nucleotide polymorphisms (SNPs) following Harris (2015), that is, SNPs that were segregating in the population of interest but that were fixed for the ancestral allele in all other populations (as singletons alone often suffer from high sequencing and variant calling errors; Han et al. 2013). These population-specific SNPs were categorized by their trinucleotide sequence context—including the mutated nucleotide and the 5′ and 3′ flanking ancestral nucleotides, leading to 192 triplets (or 96 triplets if strand complements were combined); the mutation spectrum of a population simply reflects the distribution of these categories. Thus, for each simulation, the distributions of each possible mutation type (*n* = 192) were determined based on the population-specific mutations (supplementary fig. S2, Supplementary Material online) for both the whole population as well as each subsampled population. Differences between the distributions of each mutation type were calculated by comparing the subsamples to the full population, and the sum of the differences for each mutation type per sample size was determined to obtain the total difference (fig. 1*C* and *D*).

### Simulations: Fine-scale (Local) Mutational Landscapes

In order to determine the power to identify a population-specific change in a single mutation type (such as TCC > T) at the fine-scale, 100 distinct 1 Mb regions were simulated for each species, each based on a randomly selected autosomal segment of ancestral nucleotide sequence obtained from the species-specific reference assembly. In contrast to the broad-scale (global) model above, fine-scale (local) regions were simulated using a Jukes–Cantor mutational model (i.e., an equal probability of mutation from and to every nucleotide state) with a change in mutation rate for a single mutation type. Specifically, for humans, we drew on the relative rates highlighted in Aikens et al. 2019 (see their table 1; as well as Harris 2015; Harris and Pritchard 2017; Mathieson and Reich 2017), whereas, for chimpanzees, we selected fold differences to cover the range of percent differences previously reported in humans (ranging from 0.9-fold to 2.0-fold), keeping the overall mutation and recombination rates at 10^−8^ per base pair per generation. To assess how many individuals are required to accurately identify a population-specific shift in a single mutation type at the 1 Mb scale, each population was then subsampled to sizes ranging from two to *N* individuals, drawing five independent replicates per subsample.

**Fig. 2.**
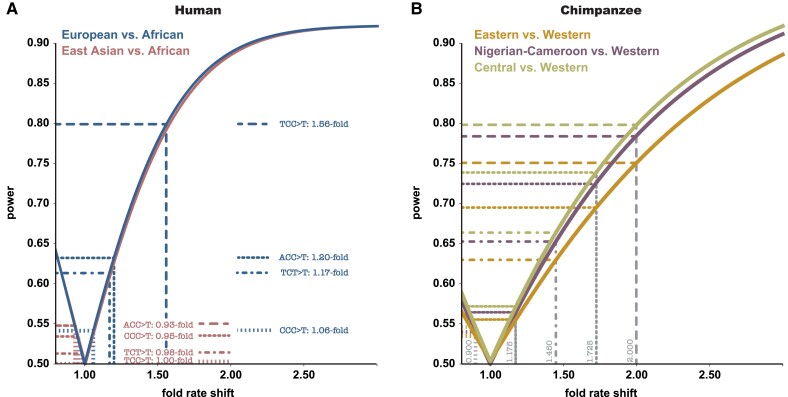
Power to detect shifts in the mutation spectrum at the fine-scale. Two-proportion *Z* tests were used to assess the power to detect a shift of a single mutation type in the mutation spectrum among (*A*) human and (*B*) chimpanzee populations at the fine-scale (1 Mb) using a sample size of 50 individuals as an example. (*a*) Depicted are relative mutation rate shifts in European (blue) as well as East Asian (pink) relative to African populations highlighted in Aikens et al. (2019) (see their table 1, as well as Harris 2015; Harris and Pritchard 2017; Mathieson and Reich 2017). (*B*) As for (*A*) but for chimpanzee populations (yellow: Eastern chimpanzees, purple: Nigerian-Cameroon chimpanzees; green: Central chimpanzees—relative to Western chimpanzees). For chimpanzee populations, relative mutation rate shifts were selected to cover the range previously observed in human populations.

Mutation-type counts—that is, the number of counts of a given category (“successes”) from among the total number of segregating private alleles (“trials”)—were assumed to behave binomially within each population. Assuming that the expected proportion of each mutation type is 1/*c*, where *c* is the number of mutational categories (i.e., 192 or 96 if strand complements were combined; Hwang and Green 2004), we considered a scenario in which a mutational shift occurred at a single mutation type in one of the two populations being compared. Two-sample *Z* tests were used to assess the power to detect such a population-specific rate change in a particular mutation type given the populations' particular demographic histories and available sample sizes (*n*1 and *n*2). Specifically, we calculated the test statistic as

$$Z=\;\frac{\;p2-p1}{\sqrt{\;p^{*}(1-\;p^{*})\left(\frac{1}{n1}+\frac{1}{n2}\right)}}$$

where $p^{*}$ is the proportion of “successes” in the pooled sample, calculated as

$$p^{*}=\;\frac{n1p1+n2p2}{n1+n2}$$

and *p*1 and *p*2 are the proportions of mutation types in the two populations being compared. With this, we tested the hypothesis that the difference between proportions was 0. As we assumed that the population affected by the mutational shift is unknown (as is the case in empirical data), we considered: (1) a mutational shift in population 1 (by keeping the expected proportion of each mutation type as 1/*c* in population 2, and introducing a shift in mutation rate for a single mutation type in population 1 using the relative rates provided by Aikens et al. 2019), or (2) a mutational shift in population 2 (keeping *p*1 constant and varying *p*2). Power was estimated as the two-tailed deviation assuming normality, averaged across population replicates, and the minimum reported (fig. 2 and supplementary figs. S3–S5, Supplementary Material online).

## Results and Discussion

Variation in mutational spectra among populations and species may be caused by a variety of factors, including population-specific differences in evolutionary processes (e.g., the extent of genetic drift, as modulated by population history), biological mechanisms (e.g., molecular processes related to DNA replication and repair), exposures to environmental mutagens (e.g., UV light), as well as technical artifacts owing to the experimental study design (Harris 2015; Anderson-Trocmé et al. 2020; and see reviews of Baer et al. 2007; Pfeifer 2020). As genomic data sets for nonmodel organisms remain limited, particularly for nonhuman hominoids, we sought to assess the robustness of mutation spectrum analyses with varying sample sizes under different demographic histories in order to help guide future investigations into the causes and consequences of changes in the mutational landscape among primates.

For the purpose of illustration, data sets of three human (African, East Asian, and European) and four chimpanzee (Nigerian-Cameroon [*P. t. ellioti*], Eastern [*P. t. schweinfurthii*], Central [*P. t. troglodytes*], and Western [*P. t. verus*]) populations were simulated as a case study, according to the demographic models previously inferred by Gutenkunst et al. (2009) (fig. 1*A*) and Prado-Martinez et al. (2013) (fig. 1*B*), respectively. In our model, we assume that mutations are context specific, independent of each other, and occur at a constant rate (in other words, we expect the same number of de novo mutations per *N_e_* generations in each population). Following Harris (2015), population-specific mutational spectra were obtained from our simulated data sets by categorizing population-specific SNPs according to their trinucleotide sequence context (including the mutated nucleotide and the 5′ and 3′ flanking ancestral nucleotides). We further assume that variation in the mutation spectrum is deterministic (i.e., different 5′ and 3′ flanking ancestral nucleotides affect the probability of a mutation from one nucleotide to another). For a single mutational category, probabilities can thus be treated as binomial. Comparative genomic analyses of population-specific mutational landscapes require comparisons of independent samples from two binomial distributions with possibly different means. The power to ascertain differences in these means is a function of the variance of each distribution which, in turn, depends on the number of private alleles sampled from each population (i.e., those alleles that have arisen independently in each population since their split). Importantly, the variance will thus be influenced by the demographic history of the populations in question.

In general, populations with historically relatively stable population sizes (such as African human populations or Western and Nigerian-Cameroon chimpanzee populations) require fewer individuals for the subsampled mutational spectra to resemble that of the entire population at the broad-scale, compared with those populations that have experienced strong bottlenecks followed by subsequent expansions (such as Asian and European human populations; fig. 1*C* and *D*, and see supplementary fig. S1, Supplementary Material online for basic “constant,” “decline,” and “growth” demographic models). As expected, larger sample sizes are also required, for example, in populations with high rates of migration (such as Eastern chimpanzee populations) due to a larger sampling variance. As a consequence, the limited sample sizes currently available for common chimpanzees (4 Central, 4 Western, 10 Nigerian-Cameroon, and 6 Eastern chimpanzees) will result in a poor representation of the population-specific broad-scale mutational landscapes (with ≥5% differences compared with the full population observed at the chromosomal scale; fig. 1*D*). This dependence of mutational spectra on population history highlights the importance of considering the covariance of mutational categories when ascertaining mutational signatures across the genome, particularly when using aggregate techniques such as principal component analysis.

In their recent analysis, Goldberg and Harris (2022) suggested that, with a few notable exceptions, compartment-specific mutational landscapes have remained conserved over long evolutionary time scales (i.e., between closely related hominoids). This motivated us to assess the effects of sampling on the ascertainment of shifts in binomial probability associated with individual mutation categories at small (1 Mb) genomic scales. Confirming previous results (Harris 2015; Harris and Pritchard 2017; Mathieson and Reich 2017), power is high to detect the most prominent signature—a mutational shift in TCC > T mutations between European and African populations, even at the fine-scale (supplementary fig. S3, Supplementary Material online, bottom left). In contrast, for the weaker signals reported in humans (ACC > T, TCT > T, and CCC > T; see table 1 in Aikens et al. 2019), sample sizes up to 50 individuals are insufficient (<30% power) to distinguish local changes in the mutation spectrum among populations (supplementary figs. S3 and S4, Supplementary Material online). Similarly, rate changes of <1.5-fold are challenging to identify among chimpanzee populations (supplementary fig. S5, Supplementary Material online, top three panels). However, even at sample sizes of 10 individuals per population, the power to identify local shifts in the mutation spectrum increases to >50% and >75% at 1.725-fold and 2.0-fold differences, respectively (supplementary fig. S5, Supplementary Material online, bottom two panels). More generally, even at larger sample sizes (e.g., 50 individuals), the power to detect shifts in the mutation spectrum at the fine-scale ranges widely, between 51.6% and 85.4% for humans (fig. 2*A*) and 53.5% and 79.5% for chimpanzees (fig. 2*B*).

## Conclusion

Sample size and population history can assert a strong influence on the observed mutational spectra. As a consequence, prior to such evolutionary genomic analysis, simulations are a highly useful tool to quantify biases, power, and false-positive rates (see also Johri et al. 2022). Specifically, as shown in our case study, the analyses here presented are necessary to (1) quantify the magnitude and scale of shifts in mutational spectra that can be reliably inferred among populations and species given a particular data set, and (2) determine the minimal sample size needed for a robust inference at a specific magnitude and genomic scale. As such, the results presented here will not only be directly informative for future mutational analyses in human and chimpanzee populations, but the described simulation and analysis framework may also be readily replicated for the study of alternative populations and species. Importantly, given the multiple contributing factors, such analyses will be required for each new population and data set under study.

## Supplementary Material

evad019_Supplementary_Data

## Data Availability

To enable reproducible research, all scripts necessary for simulation and analysis are publicly available on GitHub (https://github.com/PfeiferLab/mutational_spectra). Simulations were based on nucleotide sequences obtained from species-specific reference assemblies available in NCBI GenBank (T2T-CHM12 v.2.0 for humans [accession number: CP068257; Nurk et al. 2022] and panTro6 for chimpanzees [accession number: CM009259; Kronenberg et al. 2018]).
