# Supplementary material for: The Impact of Sample Size and Population History on Observed Mutational Spectra: A Case Study in Human and Chimpanzee Populations
Source: Genome Biol Evol. 2023 Feb 15;15(3):evad019. doi: 10.1093/gbe/evad019 (PMC9989333; doi:10.1093/gbe/evad019)
Supplement: evad019_Supplementary_Data [file evad019_supplementary_data.docx]

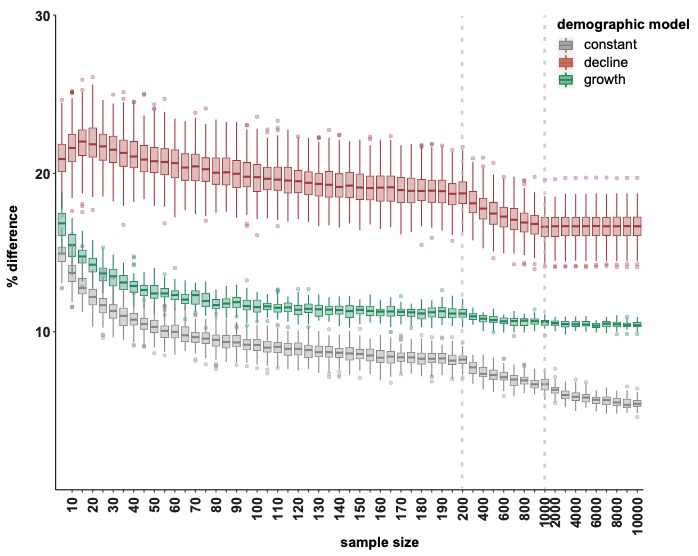


**Supplementary Figure S1**. **Effect of sample size on broad-scale mutational spectra under three basic demographic models**. Comparison of the mutational spectra between whole populations at a constant size (gray), experiencing a decline (red) or expansion (green), each consisting of an order of magnitude change in size, with populations sub-sampled with replacement to (i) 5 to 200 individuals (in increments of 5), (ii) 300 to 1,000 individuals (in increments of 100), and (iii) 2,000 to 10,000 individuals (in increments of 1,000) at the broad (chromosomal) scale. Comparisons were performed by calculating the sum of the differences in the distributions of each mutation type between the sub-samples and the whole population. Dotted lines indicate changes in sub-sampling scheme.


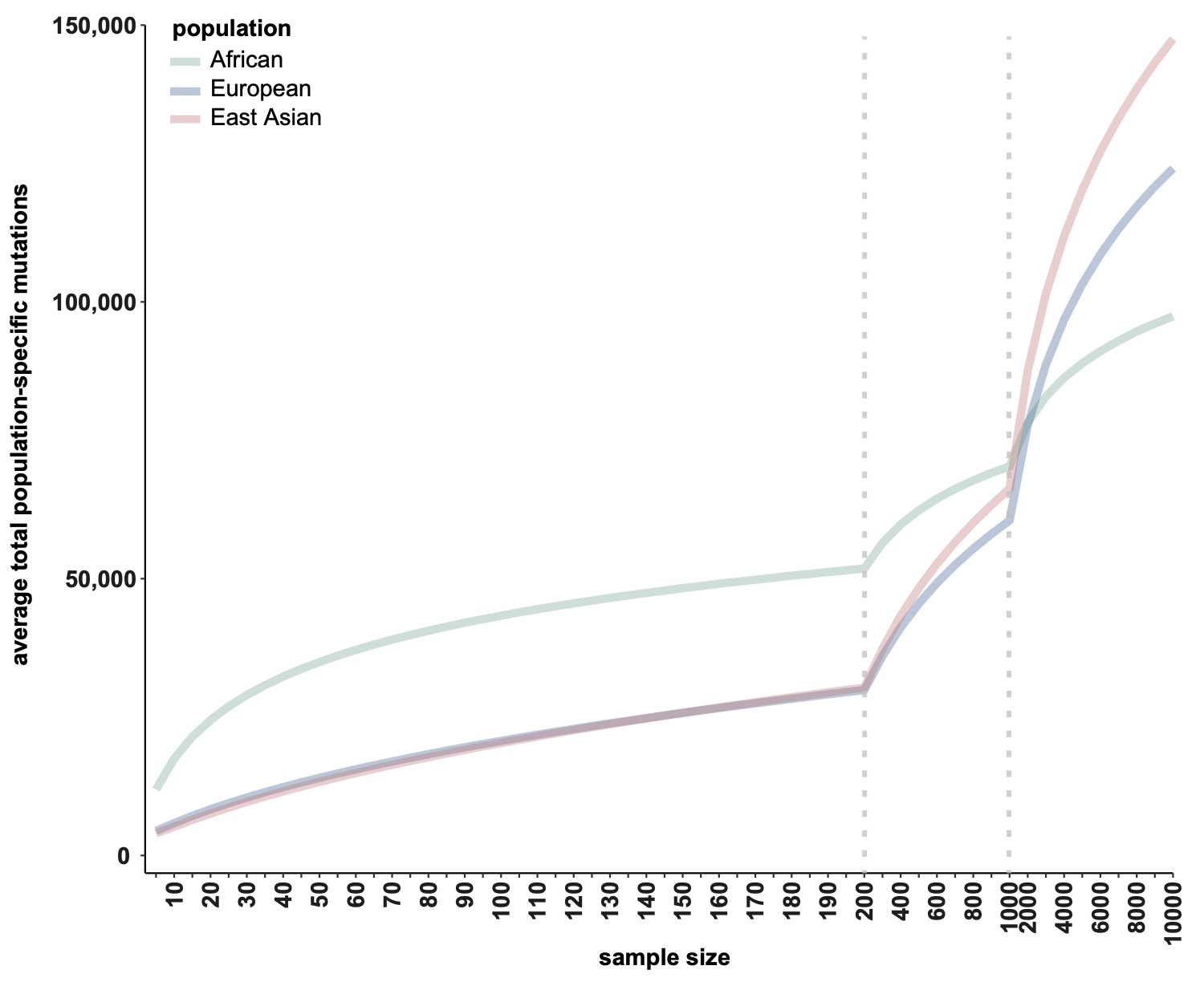


**Supplementary Figure S2**. Average number of population-specific mutations per sample size.

**Supplementary Figure S3**. Power to distinguish fine-scale mutation spectrum rate changes between African (x-axes) and European (y-axes) populations at different sample sizes (0 to 50 individuals). Rate changes among human populations were taken from Aikens *et al.* 2019 (see their Table 1). Power ranges are color-coded, with black indicating no power, dark colors indicating lower power, and light colors indicating higher power. Please note the change in scale for the TCC>T mutation in the bottom right panel.

**Supplementary Figure S4**. Power to distinguish fine-scale mutation spectrum rate changes between African (x-axes) and East Asian (y-axes) populations at different sample sizes (0 to 50 individuals). Rate changes among human populations were taken from Aikens *et al.* 2019 (see their Table 1). Power ranges are color-coded, with black indicating no power, dark colors indicating lower power, and light colors indicating higher power.

**Supplementary Figure S5.** Power to distinguish fine-scale mutation spectrum rate changes between Western (x-axes) and Nigerian-Cameroon (left) / Central (middle) / Eastern (right) chimpanzee populations (y-axes) at different sample sizes (0 to 50 individuals). Rate changes among chimpanzee populations were selected to match the range reported in humans (Supplementary Figures S1 and S2).
